# Unusual Manifestation of Toscana Virus Infection, Spain

**DOI:** 10.3201/eid1502.081001

**Published:** 2009-02

**Authors:** Sara Sanbonmatsu-Gámez, Mercedes Pérez-Ruiz, Begoña Palop-Borrás, José María Navarro-Marí

**Affiliations:** Hospital Universitario Virgen de las Nieves, Granada, Spain (S. Sanbonmatsu-Gámez, M. Pérez-Ruiz, J.M. Navarro-Marí); Hospital Clínico Universitario San Cecilio, Granada (B. Palop-Borrás)

**Keywords:** Toscana virus, chronic infection, exanthema, encephalitis, letter

**To the Editor:** Toscana virus (TOSV) causes acute meningitis and meningoencephalitis in Mediterranean countries (*1*). In Spain, neurologic TOSV infection has been reported since 1988. All cases have been self-limited aseptic meningitis (*2*). Since 2003, we have routinely investigated TOSV in cerebrospinal fluid (CSF) specimens from patients with suspected viral meningitis and encephalitis by using cell culture and reverse transcription–PCR (RT-PCR) (*3**,**4*). Also, as part of a regional project (05/305, Consejería de Salud, Junta de Andalucía, Spain), we investigated TOSV in mild nonneurologic syndromes by detection of immunoglobulin (Ig) M against TOSV by using enzyme immunoassay (Diesse Diagnostica Senese S.p.A, Siena, Italy). From May through September of 2006 and 2007, a total of 358 serum samples were randomly selected from patients for whom microbiologic determinations had been requested to investigate febrile illnesses.

As a result of these virologic and serologic surveys, we detected 7 cases of TOSV infection. Mild aseptic meningitis developed in 4 patients; in 3 patients, the infection had an atypical manifestation, as described below.

Patient 1, a 45-year-old man, was referred to the Hospital Universitario Virgen de las Nieves in September 2004 with confusion and a temperature of 39°C. He had had a splenectomy 20 years before, and in 2002, he had received a kidney transplant after renal failure resulting from meningococcemia. On admission, the patient was receiving chronic immunosuppressive treatment. Ten days after admission, he had tonic-clonic seizures. Aphasia and paresis developed after an ictus of the left hemisphere, and his level of consciousness decreased rapidly. Treatment with corticosteroids was initiated because vasculitis was suspected. The patient responded to treatment, and 2 months after admission, he was discharged. Four months later, he still had impaired speech and paresis. Lymphocytic pleocytosis, a normal glucose level, and elevated protein levels were observed in CSF samples taken during the 2-month period of hospitalization. Bacterial and fungal cultures, as well as results of PCR for enterovirus, herpes simplex virus (HSV), and varicella-zoster virus (VZV), were negative in CSF specimens taken at admission and 1 month later. TOSV was detected by cell culture and nested RT-PCR in both samples (*3*). Anti-TOSV IgG was not detected in serum samples obtained on days 1 and 10; 5 months later, a borderline result was obtained. Anti-TOSV IgM was not detected on day 1 but was detected on day 10; 5 months later, anti-TOSV IgM was detected. Sequence analysis of amplified fragments from L and S segments (GenBank accession nos. FJ356705 and FJ356706, respectively) indicated 95%–98% homology with sequences from Spanish TOSV strains (*3*) and 84% homology with Italian reference strain ISS Phl.3.

Patient 2, a 54-year-old man, was admitted to a regional hospital in Granada Province in November 2007. He was confused and agitated, and he reported having fever and headache 2 days before. On admission, he was receiving treatment with corticosteroids for Crohn disease. Analysis of the CSF specimen showed lymphocytic pleocytosis, a normal glucose level, and increased protein levels. Results of PCR for HSV, VZV, and enterovirus were negative. TOSV was detected in the CSF sample by cell culture and real-time RT-PCR (*4*). The patient was treated with antimicrobial drugs and acyclovir. He recovered and was discharged 3 weeks after admission. One month later, he returned with paresis and aphasia, secondary to an ischemic stroke located in the left hemisphere. IgG and IgM antibodies against TOSV were detected in a serum sample obtained at that time. The patient was discharged 1 week later with slight aphasia.

The most relevant common signs observed in patients 1 and 2 were the ischemic complications. Few cases of complicated encephalitis with sequelae caused by TOSV have been described (*5**,**6*). Moreover, to our knowledge, persistent neurologic TOSV infection has not been reported. The immune status of these patients probably influenced the clinical outcome in both patients and the delayed serologic response in patient 1.

Patient 3, a 41-year-old woman, sought treatment for exanthema at her health care center in July 2004. Test results for IgM antibodies against rubella, parvovirus B19, and *Rickettsia conorii* were negative. Specific anti-TOSV IgM was detected. The infection was self-limited, and no signs of neurologic involvement were associated with the rash. This was the only case of anti-TOSV IgM detection in 358 serum specimens analyzed from patients with nonneurologic syndromes. Although this finding is not conclusive, it suggests that TOSV infection might be involved occasionally in other mild syndromes. Two other cases of TOSV infection without neurologic involvement have been reported elsewhere: febrile erythema in Italy (*7*) and an influenza-like illness in southern France (*8*).

The unusual manifestations of TOSV infection reported here occurred in persons from rural areas within Granada Province, where seroprevalence rates have been shown to be higher than in urban areas (*3*). These data provide more information about this arboviral infection. Atypical TOSV infection could occur particularly in areas where the virus is endemic.
